# Leveraging multiple genomic data to prioritize disease-causing indels from exome sequencing data

**DOI:** 10.1038/s41598-017-01834-w

**Published:** 2017-05-11

**Authors:** Mengmeng Wu, Ting Chen, Rui Jiang

**Affiliations:** 1 MOE Key Laboratory of Bioinformatics; Bioinformatics Division and Center for Synthetic and Systems Biology, TNLIST, Tsinghua University, Beijing, 100084 China; 2 Department of Computer Science, Tsinghua University, Beijing, 100084 China; 3 Department of Automation, Tsinghua University, Beijing, 100084 China

## Abstract

The emergence of exome sequencing in recent years has enabled rapid and cost-effective detection of genetic variants in coding regions and offers a great opportunity to combine sequencing experiments with subsequent computational analysis for dissecting genetic basis of human inherited diseases. However, this strategy, though successful in practice, still faces such challenges as limited sample size and substantial number or diversity of candidate variants. To overcome these obstacles, researchers have been concentrated in the development of advanced computational methods and have recently achieved great progress for analysing single nucleotide variant. Nevertheless, it still remains unclear on how to analyse indels, another type of genetic variant that accounts for substantial proportion of known disease-causing variants. In this paper, we proposed an integrative method to effectively identify disease-causing indels from exome sequencing data. Specifically, we put forward a statistical method to combine five functional prediction scores, four genic association scores and a genic intolerance score to produce an integrated *p*-value, which could then be used for prioritizing candidate indels. We performed extensive simulation studies and demonstrated that our method achieved high accuracy in uncovering disease-causing indels. Our software is available at http://bioinfo.au.tsinghua.edu.cn/jianglab/IndelPrioritizer/.

## Introduction

Recent developments of high-throughput DNA sequencing technology^1^ and computational methods for sequencing data analysis^2^ have enabled the effective detection of genetic variants in the whole genome and provided a great opportunity to dissect genetic basis of not only Mendelian diseases^3, 4^ but also complex diseases^5^ and cancers^6^. Typically, in a disease study, a crowd of genetic variants are tested for enrichment in disease cases against normal controls, and variants showing significant enrichment are hypothesized to be disease-related. Further functional or biological experiments are needed to validate these selected variants and establish causal relationships. Despite of being successful in practice, such traditional strategy faces several challenges, such as substantial statistical penalty induced by the large number of candidate variants, e.g. tens of thousands variants in exome and several millions of variants in whole-genome, and the diversity of genetic variants, e.g. SNV (single nucleotide variants), indel (micro-insertion or micro-deletion), SV (structural variants), etc. In ideal settings, these problems can be solved in some degree by increasing sample size, which can provide more statistical power for the discovery of disease-related variants. However, the increase of cost and time coupled with the increase of sample size often prohibits large-scale sequencing in most disease studies. In contrast, computational prediction of functional effects of genetic variants can filter out neutral variants and significantly reduce the number of candidates, and thus attracts much attention in recent years.

Many methods have been proposed for predicting functional effects of SNV, such as SIFT^7^ and PolyPhen2^8^ for coding regions, and CADD^9^ for noncoding regions. These methods can be roughly categorized into two groups, those utilize conservation of DNA or protein sequence across species to measure disruptive effects of particular mutations, and those directly build machine learning classifiers by using known causal and neutral SNVs as training data. These methods are applied to all possible coding SNVs to derive pre-computed prediction scores, which are collected in public databases, such as dbNSFP^10^, ANNOVAR^11^ and dbWGFP^12^. These prediction scores are used frequently in sequencing-based disease studies recently, and show great utilities for SNV analysis. However, these methods have no specificity for diseases due to absence of phenotype information, and substantial candidates may remain after applying various filters. To overcome this limitation, several methods are proposed recently to incorporate phenotype information to prioritize disease-causing SNVs^9, 13–16^, showing promising performance compared with traditional methods. Besides SNVs, indels are recognized to be important by an increasing number of studies and a growing number of indels are discovered to be disease-causing. As of February 2017, nearly 40,000 indels are collected in the HGMD database^17^ (professional version). Although several methods have been developed for predicting functional effects of indels^18–22^, none of them considers the incorporation of phenotype information. To fill the gap, we proposed a method integrating phenotype information and functional effect predictions to prioritize indels from exome sequencing data.

Specifically, our method integrates five indel functional prediction scores, including CADD^23^, VEST^20^, SIFT^18^, DDIG^19, 24^ and PinPor^22^, four genic association scores derived from four different genomic data, including gene expression^25^, protein-protein interaction^26^, gene ontology^27^ and transcriptional regulation^28^, and a genic intolerance score named RVIS^29^. We transform each functional prediction score and RVIS score into a *p*-value by comparing it against the corresponding empirical null distribution. For each genic association score, we build a two-layered network, consisting a disease network, a gene network and known associations between diseases and genes, then perform random walk simulation procedure to infer association strength between given disease and query genes^30^. We also build empirical null distributions for genic association scores and transform these scores into *p*-values. Finally, we integrate these *p*-values into an integrated *p*-value by Fisher’s method with dependence correction. The integrated *p*-value thus provides a means for prioritizing indels.

We collected data from such public databases as HGMD^17^, 1000 Genomes Project^31^ and Exome Sequencing Project^32^, and we conducted a simulation studies to validate the effectiveness of our method. In simulation studies, each causal indel was spiked into the exome of a normal individual, and our method was applied to uncover the spiked causal indel. The simulation studies supported the effectiveness of our method for prioritizing causal indels and the robustness in the presence of missing data. In the future, we may further incorporate more functional genomic data into our method and extend it to more types of variants.

## Results

### Overview of our method

**Figure 1:**
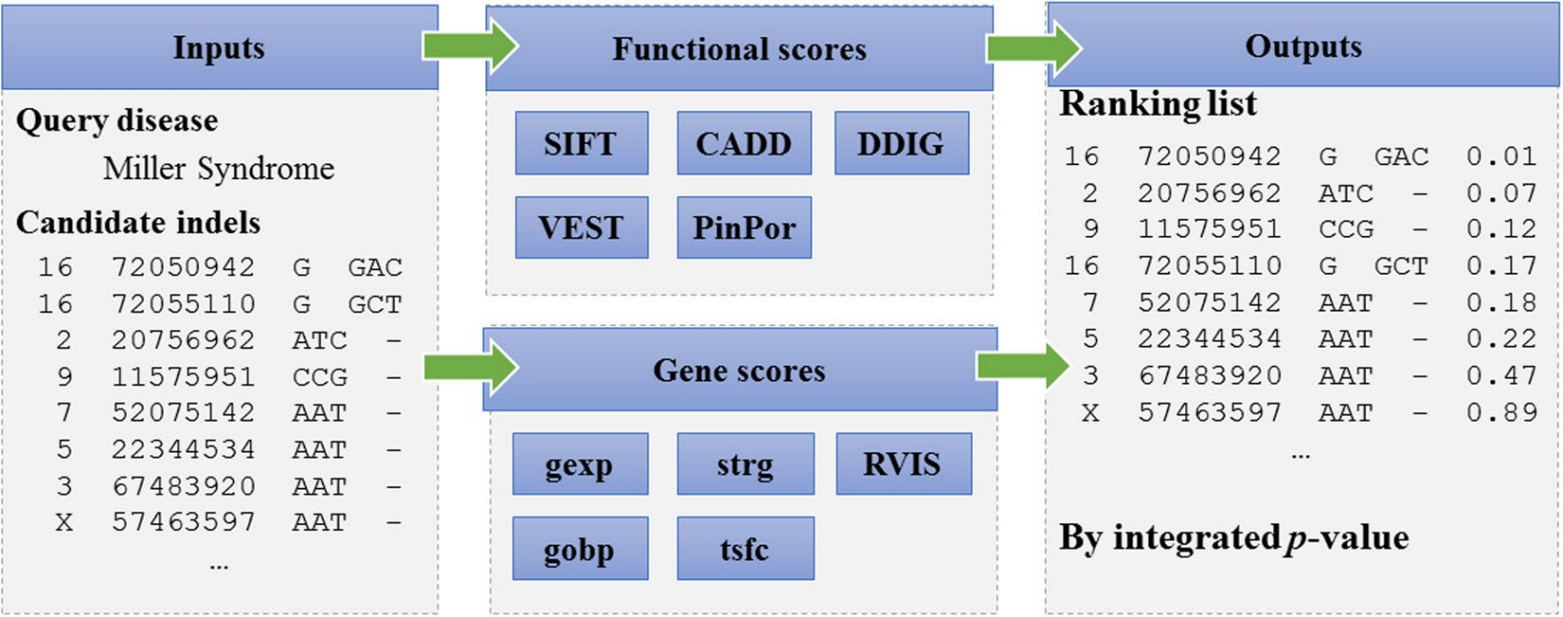
Schematic overview of our method. Our method takes a list of candidate indels and a disease of interest as input and outputs a prioritized list according to the likelihood of causing the disease. For each indel, we extract five kinds of functional scores, four kinds of association scores and RVIS score as genomic features. Each genomic feature is transformed into a *p*-value and we use Fisher’s method with independence correction to integrate these *p*-values into an integrated *p*-value, which is used to prioritize candidate indels.

As depicted in the overview paradigm (Fig. 1), our method takes a list of candidate indels and an OMIM^33^ identifier for disease of interest as input and produces a ranking list of the candidates as output. To achieve this goal, we first extract for each indel five functional prediction scores, including SIFT^18^, PinPor^22^, CADD^21^, DDIG^19^ and VEST^20^, from their corresponding websites. Because these scores are different from each other in such factors as training data, prediction method, numeric scales and so on, we transform these scores into *p*-values (detailed in “Methods”), which provides a unified representation of functionally damaging effects of candidate indels. Then, we quantify the association strength between genes hosting candidate indels and the disease of interest through a random walk model on a two-layered phenotype-gene network (detailed in “Methods”). We use four types of genomic data to construct the network, including gene expression^25^, gene ontology^27^, protein-protein interaction^34^ and transcriptional regulation^28^, and thus we obtain four types of association scores. We also transform these association scores into *p*-values. Besides, we incorporate the RVIS score^29^, which quantifies genic intolerance, and transform it into a *p*-value. Consequently, for each indel, we obtain five *p*-values for the functional prediction scores, four *p*-values for the association scores and one *p*-value for the RVIS score. Next, we use Fisher’s method with dependence correction to integrate these *p*-values into an integrated *p*-value, which quantifies the statistical strength of each indel being causative for the given disease. Finally, we prioritize candidate indels according to their *p*-values, with indels with small *p*-values ranked in top positions, indicating that they are more likely to be causal.

### Performance in simulation studies

To validate our method for prioritizing disease-causing indels, we conducted the following simulation studies. We first obtained positive samples by extracting from the HGMD database coding indels with genomic length ranging from 1 bp to 20 bp, also called micro-deletions and micro-insertions. For each of these causal indels, we mapped the text description of the annotated disease to an OMIM identifier. To ensure high data quality, we require that such a mapping must satisfy at least one of the following criterions: (1) the disease descriptive text in HGMD exactly matched that of the OMIM record; (2) the same causal variants, either in DNA sequence format or rsid, were shared by the HGMD disease and the OMIM record; (3) the same pubmed ID was shared by the HGMD disease and the OMIM record. Although such rules removed many low quality mapping, one-to-many or many-to-many mapping could still occur due to pleiotropic effects of some variants. We therefore focused on only diseases with one-to-one mapping in the HGMD and OMIM databases and discarded indels with multiple mapped OMIM identifiers. With the above rules, we obtained a set of indels with high quality and uniquely mapped OMIM identifier, and these indels could serve as the gold-standard for validating our method. We then retrieved negative samples from two public sequencing project data, including 1000 Genomes Project (KG for short) and Exome Sequencing Project (ESP for short). Here, we followed the strategy used in VEST and retained indels with minor allele frequency (MAF) > 1% in the African American subpopulation. The collected indels were categorized into four subtypes: (1) nonframeshift deletion (ND for short), (2) nonframeshift insertion (NI for short), (3) frameshift deletion (FD for short), and (4) frameshift insertion (FI for short). The detailed summary statistics about data used in the simulation studies were presented in Table 1, which suggested obvious enrichment of frameshift indels in the HGMD dataset, when compared with the KG and ESP datasets (Fisher’s exact test, *p*-values < 2.2 × 10^−16^). This may partly be attributed to severe consequence induced by frameshift indels since they can disrupt all amino acids after indels while nonframeshift indels only alter several amino acids. The coverage of each functional scores for data used in simulation studies was presented in Table 2, which showed that coverage varied across different datasets and the integration of multiple scores in deed helped to improve the overall coverage.

**Table 1:** Summary about data used in the simulation studies.

Data source	ND	NI	FD	FI
KG	531	319	380	210
ESP	1,546	321	1,164	835
HGMD	1,228	255	9,167	3,953

Abbreviations: ND (nonframeshift deletion), NI (nonframeshift insertion), FD (frameshift deletion), FI (frameshift insertion). Each entry denotes the number of indels for each indel subtype in the three datasets.

**Table 2:** Coverage of each genomic data on the datasets used in the simulation studies.

Data	Score	ND	NI	FD	FI
ESP	SIFT	94.76%	93.76%	93.64%	94.25%
	PinPor	99.54%	99.68%	98.96%	99.04%
	CADD	99.93%	100%	99.91%	99.64%
	DDIG	95.47%	96.26%	92.26%	94.25%
	VEST	99.74%	100%	98.88%	99.40%
	RVIS	82.08%	80.68%	81.27%	70.54%
KG	SIFT	91.71%	89.96%	88.68%	85.71%
	PinPor	97.55%	97.80%	91.84%	95.71%
	CADD	100%	100%	100%	100%
	DDIG	93.22%	92.16%	84.73%	80.47%
	VEST	99.05%	99.68%	97.63%	99.52%
	RVIS	84.55%	78.68%	81.05%	73.80%
HGMD	SIFT	96.82%	94.90%	98.65%	98.20%
	PinPor	100%	100%	100%	100%
	CADD	62.78%	58.03%	68.07%	68.15%
	DDIG	48.45%	58.03%	68.07%	68.12%
	VEST	59.85%	100%	58.25%	100%
	RVIS	93.48%	87.45%	94.27%	92.84%

Each entry denotes the coverage rate of each genomic data for each indel subtype in the three datasets.

For each disease-causing indel, we spiked it into the corresponding control dataset of the same subtype and applied our method to prioritize the resulting simulated data set. Here, we conducted simulations for these four subtypes of indels separately, in order to avoid the bias of imbalance due to the obvious difference of the number of each subtype. With integrated *p*-values supplied by our method, we obtained a rank for each disease-causing indel from the final prioritization list, and derived two metrics for overall performance evaluation. First, we counted the number of disease-causing indels ranked among top 20 and referred to this criterion as TOP. Second, we defined rank ratio of each disease-causing indel by dividing its rank by the number of all indels in the dataset. The rank ratios of all disease-causing indels were then averaged to get an overall measure, called as MRR. Typically, high TOP and low MRR together indicate good performance.

**Figure 2:**
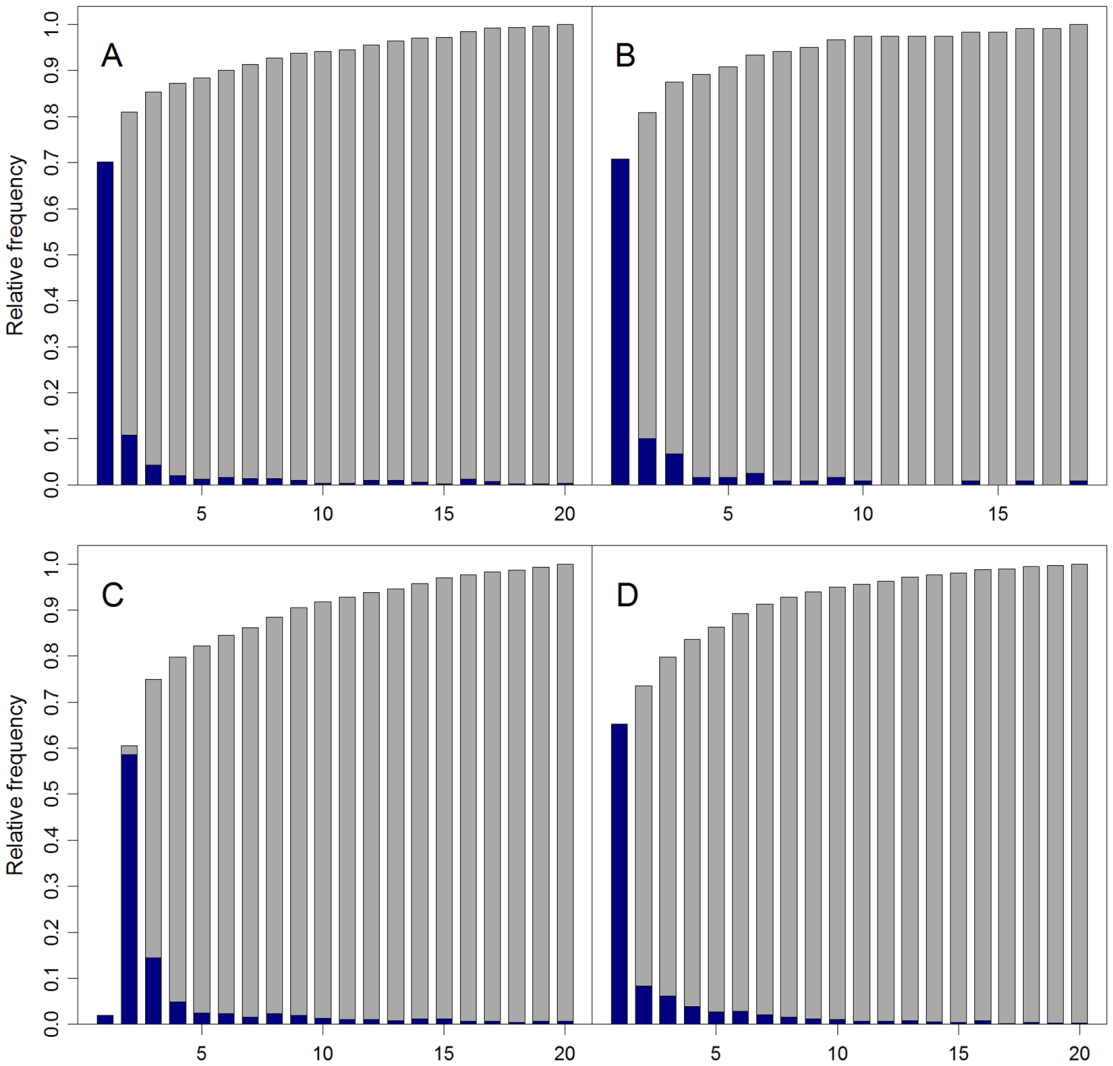
Rank histogram on simulation studies. Ranks of disease-causing indels against neutral indels, for different indel subtypes: (**A**) nonframeshift deletion; (**B**) nonframeshift insertion; (**C**) frameshift deletion; (**D**) frameshift insertion.

The rank histograms of causal indels were presented in Fig. 2, and the overall ranks of causal indels were obviously skewed towards top position. For example, over 80% of disease-causing indels were ranked in top 5 for all indel subtypes, while the corresponding numbers for random guess would be 0.9%, 1.6%, 1.3% and 2.3% respectively, significantly less than that of our method. We also observed the same trend for causal indels ranked in top 10 and top 20. This demonstrated that our method could effectively uncover the real disease-causing indels in top positions. On the other hand, the MRRs for these four indel subtypes were 1.67%, 1.06%, 3.62% and 2.19%, while MRRs of random guess would be 50%, significantly worse than our method. Both MRR and TOP metrics supported the effectiveness of our method. We also performed a prospective simulation study, in which we extracted HGMD disease-causing indels that were discovered after 2015 from the HGMD database (professional version 2016. (4) We used these indels as cases and performed the same simulation study as before, and the results were shown in Supplementary Table 1. We observed similar performance in terms of MRR and TOP, suggesting our evaluation was unbiased.

### Integration helps performance improvement

We next investigated whether integration of multiple genomic data could significantly improve prioritization performance. With the same simulation studies mentioned above, we evaluated the performance of individual genomic data for prioritization of disease-causing indels. The results were shown in Table 3, in which both MRR and TOP were evaluated for each genomic data. Here, we also considered the influence of missing data, and last position was assigned to the causal indel if the corresponding genomic data was missing. Therefore, the final performance of each genomic data relied on both its predictive power and coverage, and genomic data with high coverage and strong predictive power would have good performance.

**Table 3:** Performance of each individual genomic data and the integrated score for the simulation studies.

Score	MRR				TOP			
	ND	NI	FD	FI	ND	NI	FD	FI
SIFT	27.80%	24.16%	26.37%	25.57%	31	18	1	0
CADD	62.01%	75.12%	43.76%	42.38%	7	0	249	61
DDIG	54.72%	46.44%	56.43%	40.89%	455	102	2,334	1,344
VEST	43.40%	3.12%	52.38%	7.08%	489	141	3,524	2,089
PinPor	39.16%	34.19%	33.71%	38.71%	49	22	90	56
RVIS	28.65%	30.95%	30.87%	31.67%	61	11	1,066	465
gobp	5.55%	3.44%	8.69%	9.15%	949	210	6,724	2,956
strg	10.78%	10.09%	14.51%	15.04%	935	196	6,843	2,943
gexp	19.53%	15.65%	24.26%	23.82%	477	117	3,670	1,547
tsfc	29.36%	28.39%	32.58%	32.06%	408	103	3,304	1,475
**Integration**	**1.67%**	**1.06%**	**3.62%**	**2.19%**	**1,082**	**231**	**7,614**	**3,445**

Each entry denotes MRR or TOP of each individual score, and the integrated score achieves the best performance.

**Figure 3:**
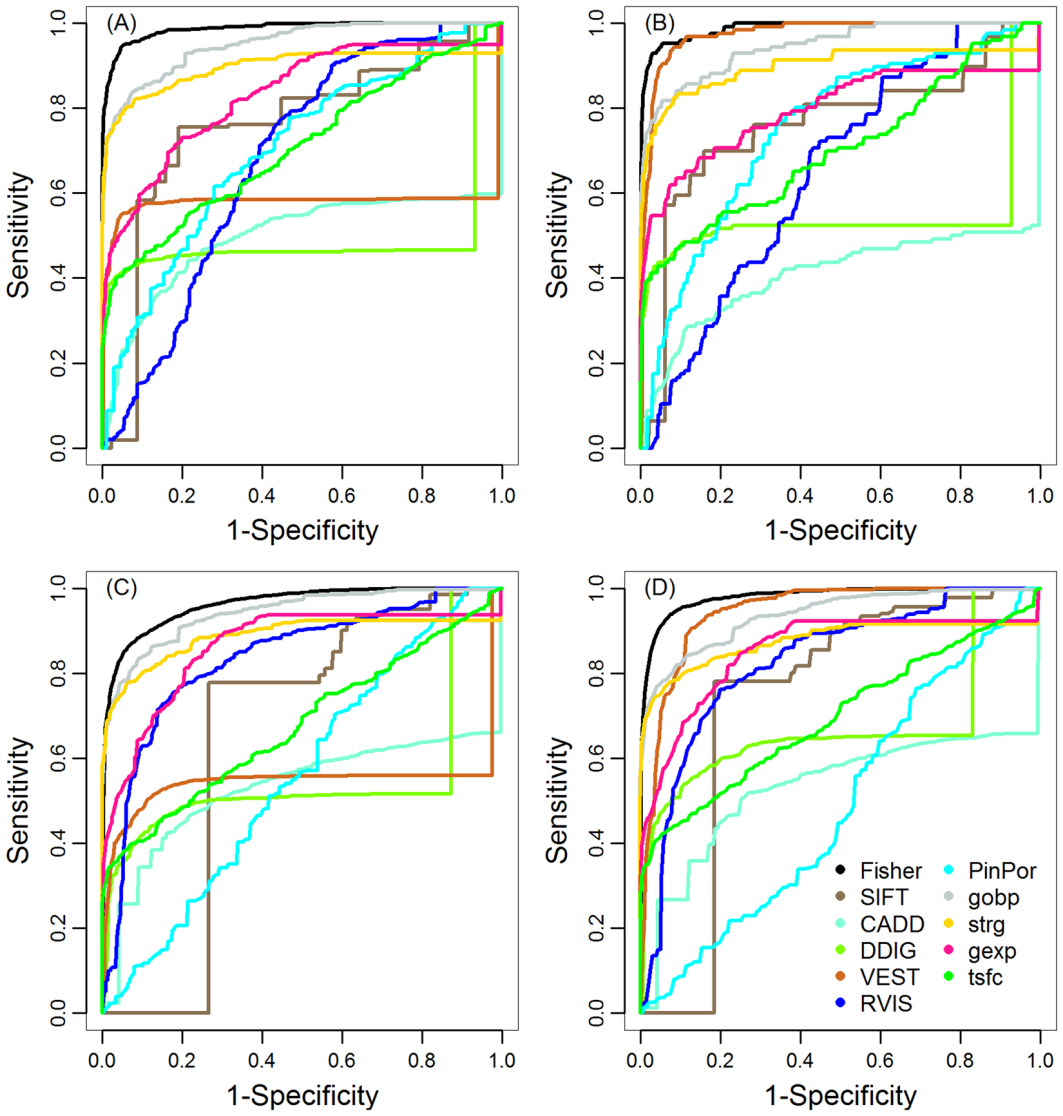
ROC curves of each individual genomic data and the integrated score. Based on results of the simulation studies, we plotted rank ROCs of each individual genomic data and the integrated score for: (**A**) nonframeshift deletion; (**B**) nonframeshift insertion; (**C**) frameshift deletion; (**D**) frameshift insertion.

From Table 3, we clearly observed that our integration method outperformed every individual genomic data alone. Specifically, the MRRs of individual genomic data ranged from 5.55% to 62.01%, 3.44% to 75.12%, 8.69% to 56.43% and 7.08% to 42.38%, while our integration method achieved MRR of 1.67%, 1.06%, 3.62% and 2.19% for the four indel subtypes, respectively. The TOPs of individual genomic data ranged from 7 to 949, 0 to 210, 1 to 6,843 and 0 to 2,956, while our integration method achieved TOP of 1,082, 231, 7,614 and 3,445, respectively. Besides MRR and TOP, we also evaluated rank ROC for performance comparison. Given a threshold for rank ratio, ranging from 0 to 1, we defined the true positive rate (TPR, also called sensitivity) as the proportion of disease-causing indels with rank ratios below the threshold and the false positive rate (FPR, also called 1-specificity) as the proportion of neutral indels with rank ratios below the threshold. By varying the threshold, we obtained a series of TPR and FPR values and plotted TPR against FPR to obtain rank ROC. From Fig. 3, we found that our method had better rank ROC than any individual genomic data.

**Figure 4:**
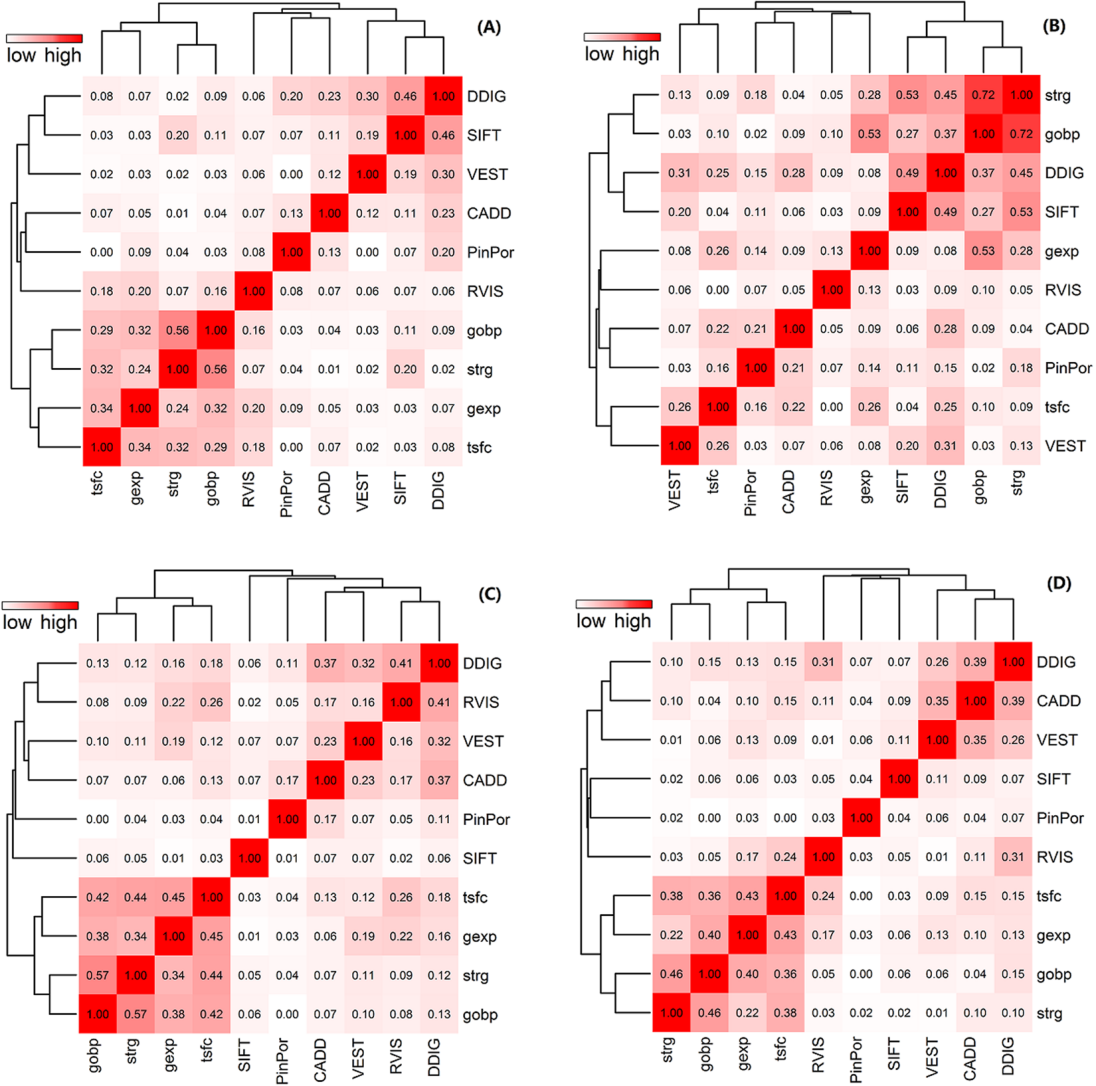
Correlations between individual genomic data. Pearson’s correlation coefficients between each pair of individual genomic data for: (**A**) nonframeshift deletion; (**B**) nonframeshift insertion; (**C**) frameshift deletion; (**D**) frameshift insertion.

The gained improvement in prioritization performance could be largely attributed to integration of diverse genomic data. These genomic data could be categorized into three groups in general, the first group of functional prediction scores, including SIFT, CADD, PinPor, DDIG and VEST, the second group of genic association scores, including gobp, gexp, strg and tsfc, and third group of the genic intolerance score RVIS. The functional prediction scores could discriminate between damaging indels and neutral indels, while it could not discriminate causal indels responsible for different diseases, because all damaging indels manifested similar effect, such as altering protein structures and functions. However, given a disease of interest, we only interested in these damaging indels responsible for the given disease. The second group of genic association scores could prioritize genes which may be associated with the disease of interest, and these genes are more likely to harbour disease-causing indels responsible for the given disease. In addition, RVIS provides genic intolerance, and genes with low RVIS scores are more likely to be disease relevant genes. We draw the correlations between these genomic data for the four different indel subtypes (Fig. 4). From this figure, we found that different correlation patterns existed for different indel subtypes, manifested as different hierarchical clustering results. In general, obvious correlations between genic association scores were observed and no correlations between these association scores and the other functional prediction scores. This reflected that association scores and functional prediction scores measured different genomic activities. Thus, these three types of genomic data sources complement with each other, and integration of them can help us prioritize real disease-causing indels.

### Comparison of different data fusion strategies

**Figure 5:**
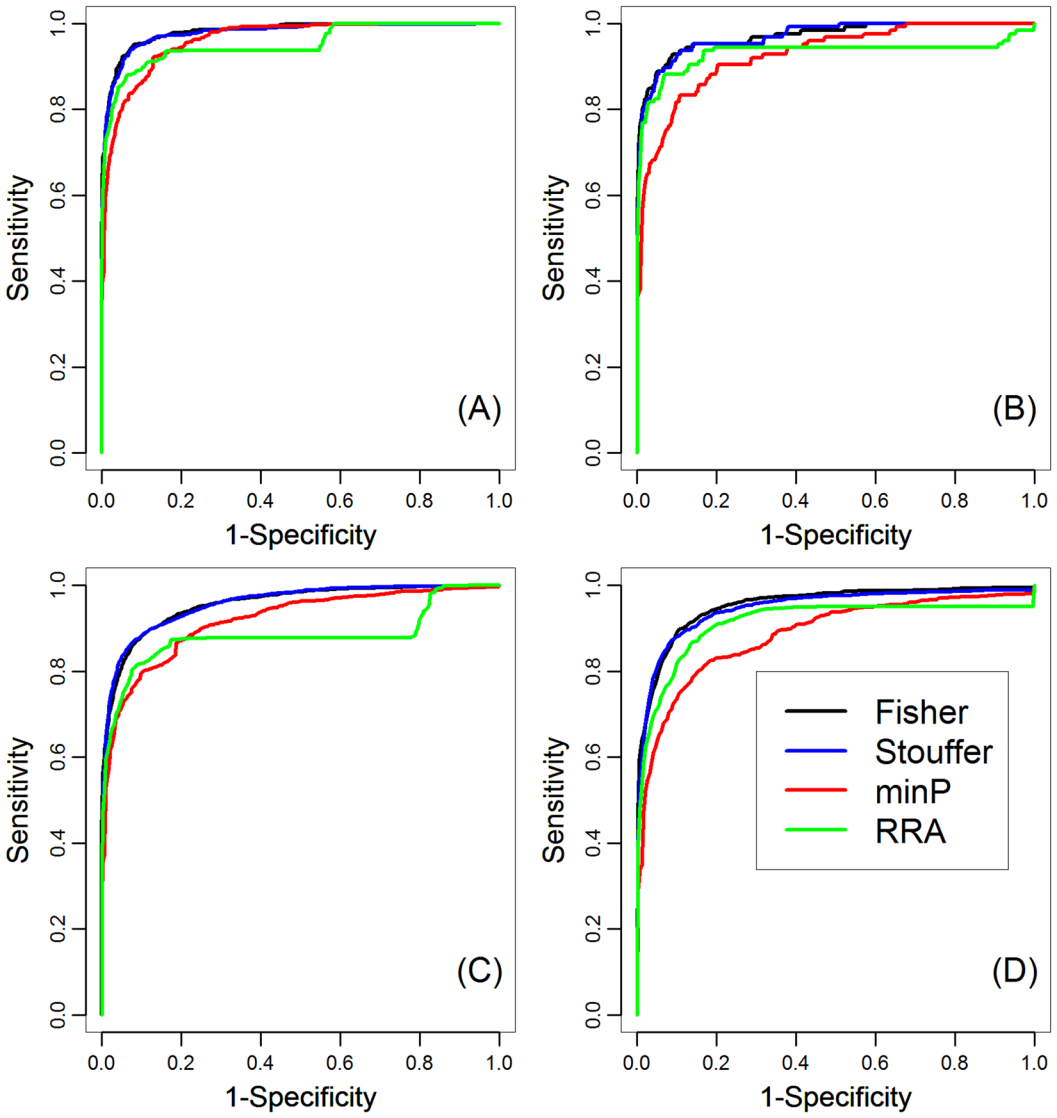
ROC curves of different data fusion strategies. Rank ROCs of four different data fusion strategies for (**A**) nonframeshift deletion; (**B**) nonframeshift insertion; (**C**) frameshift deletion; (**D**) frameshift insertion.

We adopted Fisher’s method (Fisher for short) for integration of *p*-values, which was an old but well-established and widely-used method. We also investigated the other three methods for integration of *p*-values, named minimal *p*-value (minP for short), Stouffer’s Z-score method (Stouffer for short) and Robust Rank Aggregation^35^ (RRA for short). With the same simulation studies mentioned above, we applied these three methods for disease-causing indel prioritization and evaluated the corresponding MRRs and TOPs for comparison. The results were shown in Supplementary Table 2, and we found that Fisher’s method was similar with Stouffer’s method in terms of MRRs and TOPs, which was not surprising because the two methods had close connections and nearly same statistical power asymptotically. Minimal *p*-value followed the two methods and RRA exhibited worst performance. It was worth noting that RRA was original designed for rank aggregation and its algorithm was not optimized to perform *p*-values integration. Another interesting phenomenon was that these methods have similar TOP performance in spite of different MRRs. We also compared rank ROCs of these four methods, as shown in Fig. 5, from which the same conclusion could be draw. Thus, one can pick anyone of them if only TOP performance is cared about and minimal *p*-value method shows great advantage over the others for its simplicity.

We also compared the robustness of these methods in terms of missing data, and presented the results in Supplementary Figure 1. We used the results generated from above simulation studies and categorized these indels by the number of missing functional scores, ranging from 0 to 6. We then evaluated the rank ratios of each indels in each subgroup and investigated the influence of the number of missing data on performance for each method. We observed increasing rank ratios (decreasing performance) with increasing the number of missing data, for all these methods. This was reasonable because missing data indicated missing information and posed challenge for prioritization. Stouffer’s method assigned nearly 1 (meaning last position) to several indels in all subgroups, while the other three methods avoided that. The median rank ratios for all method were near zero when the number of missing data was less than 4 and began to increase obviously after that. Stouffer’s method had the best control when the number of missing data was over four, followed by Fisher’s method and the other two methods performed worse. Generally speaking, Fisher’s method had both best performance and relatively robust controls over missing data, thus it was considered to be the most suitable method for our problem.

### Performance for personal exome

The simulation studies presented above only focused on uncovering disease-causing indels from common indels (MAF > 1%), while realistic personal exome harboured both common indels and rare indels (MAF < =1%). We randomly selected an individual (HG00096) exome from KG dataset, and draw the MAF histogram of indels from this individual in Supplementary Figure 2, from which we observed a roughly two-component mixture distribution, including common and rare components. We also investigated the functional prediction scores for the two different components, as shown in Supplementary Figure 3. There existed some difference between the distribution of functional scores of common and rare indels and rare indels tended to have high pathogenic possibility. Besides individual HG00096, we also randomly selected another nine individuals from different populations as listed in Supplementary Table 3. We assumed no disease-causing indels existed in anyone of these ten individuals owing to the healthy status of them. We then spiked each of HGMD disease-causing indels into each individual exome and prioritized the simulated exomes, which was a more difficult task than before due to the introduction of neutral rare indels. From Supplementary Table 3, we found that the MRRs of causal indels tended to be larger than before and the TOPs were also fewer than before. We compared the rank ratios of each causal indels between the two simulation studies and found it was significantly (Wilcoxon rank sum test, *p*-value < 2.2 × 10^−16^) worse in these individual exome data. Despite of difficulties presented in individual exome data, our method achieved relatively satisfactory results, as shown in Supplementary Table 3. Specifically, the average MRRs for the four indel subtypes were 2.71%, 1.99%, 4.88% and 3.35% respectively and the average TOPs were 509, 116, 3,220 and 1,434. The results on these ten individuals were similar, suggesting the robustness of our method against different genetic backgrounds. These results collectively supported our method for effectively prioritizing disease-causing indels in personal exome.

## Conclusions and Discussion

In this paper, we proposed an integrative method to prioritize disease-causing indels from exome sequencing data. Our method integrates five functional prediction scores, four genic association scores and a genic intolerance score with a statistical method. Our integration procedure mainly consists of two steps, transforming various genomic data scores into *p*-values and integrating these *p*-values with Fisher’s method. Our method enjoys several advantages, including simplicity, robustness in terms of missing data, effectiveness in terms of prioritization power and flexibility for further development. We believe that our integration methodology not only be useful for variant analysis, but also can benefit the other genomic studies involving data integration.

Despite of being effective, our method can be further improved in several aspects. First, we focus on coding indels and ignore noncoding indels. How to extend our method to be capable of analysing noncoding indels remains a topic of future research. Second, more genomic data are generated and some of them may be proved to be useful for variant prioritization, such as transcriptomic data, epigenomic data, and other phenotypic information etc. It is not difficult to incorporate other genomic data into our method, in which additional genomic data are transformed into *p*-values with appropriate procedures and these new *p*-values are integrated with existing *p*-values to obtain the integrated *p*-values. Third, we assume the correctness of variant calling in present study and ignore the quality information supplied in VCF files. It is worth noting that correct calling of indels still remains a challenge to be solved. How to incorporate quality information of indel calling into our method is another research direction of our interest.

## Materials and Methods

### Data sources

We downloaded the HGMD database (professional version 2014.3), KG and ESP databases from corresponding official websites and extracted indels satisfying criterion mentioned in simulation studies. We then extracted five types of functional prediction scores for these indels from corresponding websites: SIFT (http://sift.bii.a-star.edu.sg/), PinPor (http://watson.compbio.iupui.edu/pinpor), CADD (http://cadd.gs.washington.edu/), DDIG: (http://sparks-lab.org/ddig/), VEST (http://www.cravat.us/CRAVAT/).

We downloaded 7,719 diseases with text descriptions from the OMIM database (accessed in February 2014) and 20,327 genes from Ensemble database (accessed in March 2014). OMIM ID and Ensemble ID were used to represent disease and gene respectively in our study. With BioMart tool, we also obtained 4,368 associations between 3,709 diseases and 2,870 genes, and mappings between gene Ensemble ID and gene HGNC symbol. We downloaded the RVIS scores from its website (http://genic-intolerance.org/index.jsp).

### Transform functional scores into p-values

For each type of functional scores, we first build an empirical null distribution from either public or private databases, which puts equal probability on each data point. For a query functional score, we calculate the *p*-value as the proportion of null distribution data points with extremer (e.g. larger) scores than query score

1 $$p=\sum _{i=1}^{N}{\rm{I}}({s}_{i}\ge s)$$

where *s*_*i*_, *i* = 1, …, *N* are functional scores from the empirical null distribution, *s* the query functional score, and I(·) the indicator function. The above equation holds for scenario where larger scores denote higher damaging (CADD, VEST, and DDIG), formulation is replaced by $p={\sum }_{i=1}^{N}{\rm{I}}({s}_{i}\le s)$ if smaller scores denote higher damaging (SIFT, PinPor). For RVIS score, smaller ones denotes lower tolerance and corresponding genes have high probabilities to be disease-causing, thus we transform RVIS scores into *p*-values with $p={\sum }_{i=1}^{N}{\rm{I}}({s}_{i}\le s)$.

### Construction of disease network

To construct disease network, we first collected 10,346 concepts from the HPO database^36^ and annotations for 6,346 human diseases, which were used to construct a 10,346 dimensional numeric vector (concept vector) for each disease. Each entry of the concept vector characterizes the information of the disease relating to the corresponding HPO concept, calculated as −log(*h*_*i*_), where *h*_*i*_ is the frequency of concept *i* occurred in text description of the disease. We calculated the similarity score between every pair of disease as the cosine of the angle between concept vectors of the two diseases. We then obtained a similarity matrix for all human diseases by applying the above method to each pair of disease. In order to remove low confident edges in this network, we only retained 10 neighbouring diseases with highest similarity scores for each disease, leading to a nearest neighbour network, which we referred to as the disease network.

### Construction of gene network

We constructed four gene networks with different genomic data, including gene expression (gexp), gene ontology (gobp), protein-protein interaction (strg) and transcriptional regulation (tsfc).

#### gexp

We obtained a whole-genome gene expression profile^25^, which measured expression levels of 44,775 transcripts across 79 human tissues. Each gene was represented by a 79 dimensional numeric vector (expression vector) with each dimension denoting the expression level in the corresponding human tissue. We obtained the raw similarity scores between pairs of genes by calculating the Pearson’s correlation coefficient of their expression vectors and an exponential transformation was applied to remove noise as:

2 $${\phi }_{ab}=\exp \,[-{(\frac{1-{w}_{ab}}{\sigma })}^{2}]$$

where *φ*_*ab*_ was the transformed similarity score for gene *a* and *b*, *w*_*ab*_ the raw similarity score and *σ* the standard deviation of raw similarity scores for all gene pairs. We obtained a gene similarity matrix by applying the above method to each pair of genes and we only retained 100 neighbouring genes of highest similarity for each gene in order to remove low confident edges, leading to a final gene network (gexp).

#### gobp

We downloaded the gene ontology database^27^ (November 22, 2014) and extracted 25,616 concepts associated with biological process domain and used a 25,616 dimensional numeric vector (gene ontology vector) to characterize each gene with each dimension denoting whether the gene had the corresponding concept. We obtained the raw similarity score between each pair of genes by calculating the cosine of the angle between their gene ontology vectors and transformed these raw similarity score with the exponential transformation mentioned above. We finally obtained the gene network (gobp) with the same strategy mentioned above.

#### strg

We downloaded the STRING database^26^ (version 9.1) and extracted 403,514 interactions between 13,747 proteins, leading to a binary gene network. For each pair of proteins (e.g. *a* and *b*), we calculated the shortest path distance between them in the PPI network as *δ*_*ab*_ and rescaled this score with

3 $${w}_{ab}=1-\frac{{\delta }_{ab}}{{\rm{\max }}\,{\delta }_{a^{\prime} b^{\prime} }}$$

We also applied the exponential transformation mentioned above to this score to obtain the final similarity score, and constructed the gene network (strg) with the same strategy as above.

#### tsfc

We extracted 218 vertebrate transcription factors from the TRANSFAC database^28^ with high confident position specific scoring matrices and used the program MATCH to identify potential binding sites for each transcription factor within the 1,000 basepairs upstream of each human gene. Based on the matching results, we constructed a 218 numeric vector (binding vector) for each human gene with each dimension denoting the number of potential binding sites of the corresponding transcription factor on the gene. We calculated raw similarity score between each pair of genes as the cosine of the angle between their binding vectors. We then applied the exponential transformation on the raw similarity scores and constructed a gene network (tsfc) using the same strategy mentioned above.

### Random walk on two-layered network for disease-gene association inference

We constructed a disease-gene heterogeneous network, consisting of a disease network, a gene network, and known associations between diseases and genes, and simulated the random walk process on this network to infer the association strength of a given disease-gene pair^37^. The disease network was the one constructed as above, which encoded the similarities between diseases, the gene network was one of the four gene networks constructed as above, encoding similarities between genes, and the known links between diseases and genes were collected from the OMIM databases as mentioned above.

For a query disease of interest, a random walker started a series of moves on the disease-gene network with some initial probability *p* ^(0)^, which encoded the interested disease. At each move, the walker either restarted with probability *π* or move on with probability 1 − *π*. If moves on, the walker may switch between disease network and gene network with probability *τ* or just wander within either disease or gene network with probability 1 − *τ*. When wandering about within disease or gene network, the walker moved to one of its direct neighbours with probabilities proportional to the similarities between current node and its direct neighbours, e.g. higher probabilities moving to more similar neighbours. Over a series of iterations, the probability distribution of the walker on the network will converge to a steady probability vector *p* ^(∞)^, providing a measure of the association strength between the query disease and genes.

Mathematically, we used a triple *H* = (D, G, A) to denote the disease-gene heterogeneous network, where *D* = {d_*ij*_}_*m*×*m*_ collected edge weights of the disease network, *G* = {g_*ij*_}_*n*×*n*_ collected edge weights of the gene weights, *A* = {a_*ij*_}_*m*×*n*_ collected binary indications of disease-gene associations, and *m* and *n* the number of diseases and genes, respectively. We normalized each row of *D* to obtain a transition matrix *U* = {u_*ij*_}_*m*×*m*_, where ${u}_{ij}={d}_{ij}/{\sum }_{j=1}^{m}{d}_{ij}$ denoted the transition probability from disease *i* to disease *j*. Similarly, we derived the other three transition matrices: *V* = {v_*ij*_}_*n*×*n*_ with ${v}_{ij}={g}_{ij}/{\sum }_{j=1}^{n}{g}_{ij}$ denoting the transition probability from gene *i* to gene *j*, *R* = {r_*ij*_}_*m*×*m*_ with ${r}_{ij}={a}_{ij}/{\sum }_{j=1}^{n}{a}_{ij}$ (*r*_*ij*_ = 0 if ${\sum }_{j=1}^{n}{a}_{ij}=0$) denoting the transition probability from disease *i* to gene *j*, and *S* = {s_*ij*_}_*n*×*m*_ with ${s}_{ij}={a}_{ji}/{\sum }_{j=1}^{m}{a}_{ji}$ (*s*_*ij*_ = 0 if ${\sum }_{j=1}^{m}{a}_{ij}=0$) denoting the transition probability from gene *i* to disease *j*. We then defined matrix *T* as

4 $$T=(\begin{array}{cc}(1-\tau ){\rm{U}} & \tau R\\ \tau S & (1-\tau ){\rm{V}}\end{array})$$

and performed row-normalization to obtain overall transition matrix for the heterogeneous network as $W={\{{{\rm{w}}}_{ij}\}}_{(m+n)\times ({\rm{m}}+{\rm{n}})}$ with ${w}_{ij}={t}_{ij}/{\sum }_{j=1}^{m+n}{t}_{ij}$ denoting transition probability within the heterogeneous network.

Let ${u}^{(0)}={\{{{u}_{i}}^{(0)}\}}_{m\times 1}$ and ${v}^{(0)}={\{{{{\rm{v}}}_{i}}^{(0)}\}}_{n\times 1}$ denote the initial probabilities for the diseases and genes, respectively. We assigned equal probabilities to the neighbours of the query disease to *u* ^(0)^ and all zeros to *v* ^(0)^, assuming a completely unknown genetic basis. Let ${p}^{(0)}={({({{\rm{u}}}^{(0)})}^{T},{({{\rm{v}}}^{(0)})}^{T})}^{T}$ denotes the initial probabilities for the heterogeneous network and *p* ^(t)^ denotes the probability after *t* moves, the interaction can be formulated as

5 $${p}^{(t+1)}=(1-\pi ){{\rm{p}}}^{(t)}+\pi {p}^{(0)}$$

Repeating the interaction until *p* ^(*t*)^ becomes stable (e.g., ${\Vert {p}^{(t+1)}-{p}^{(t)}\Vert }_{2}^{2} < \varepsilon$, where *ε* is a small positive number), and we obtained the steady-state probability *p* ^(∞)^, consisting of a disease probability *u* ^(∞)^ and a gene probability *v* ^(∞)^. The gene probability can be used to infer the association strength between the query disease and genes. We set default values for parameters *τ* = 0.5, *π* = 0.5 and *ε* = 10^−4^ according to the literature^37^.

We then transformed the steady-state probabilities to *p*-values in order to facilitate subsequent integration. We simulated the random walk process for all disease-gene pairs that were not associated and obtained the distribution of steady-state probability under no association. Then we assigned a *p*-value for a query disease-gene pair as

6 $$p=\Pr ({\rm{scores}}\,{\rm{of}}\,\text{non} \mbox{-} \text{associated}\,\text{disease} \mbox{-} \text{gene}\,{\rm{pairs}}\ge {\rm{the}}\,{\rm{query}}\,{\rm{score}})$$

Here, score referred to the steady-state probability and this *p*-value quantified the probability of observing stronger association scores under no association (null hypothesis). We excluded the known link between the query disease and the query gene when inferring their association.

### Fisher’s method for *p*-values integration

We have ten different genomic data, leading to ten *p*-values through computations described as above, and Fisher’s method is used to integrate these *p*-values into an integrated *p*-value, which quantify the statistical significance of candidate indels causing the query disease.

In detail, let *p*_1_, …, *p*_*K*_ denote the *p*-values to be integrated, where *K* = 10 in our study, we then define the Fisher’s statistic as

7 $$U=\sum _{i=1}^{K}{V}_{i}$$

where *V*_*i*_ = −2 log *p*_*i*_. If independence between different sources is assumed, it is obvious to verify that $U \sim {\chi }_{2K}^{2}$ since ${p}_{i} \sim {\rm{Uniform}}(0,1)$ and $-2\,\mathrm{log}\,{p}_{i} \sim {\chi }_{2}^{2}$. However, this independence assumption usually does not hold in reality, and we assume this statistic follows a scaled chi-squared distribution with scale *η* and degrees of freedom *v*. With method of moments, we derived the matching equations as

8 $$\{\begin{array}{rcl}{\rm{E}}[\eta {\chi }_{v}^{2}]={\rm{E}}[{\rm{U}}]\, & \Rightarrow  & \eta {\rm{v}}=2K\\ {\rm{Var}}[\eta {\chi }_{v}^{2}]=\mathrm{Var}[U] & \Rightarrow  & 2{\eta }^{2}{\rm{v}}=4\sum _{k=1}^{K}\sum _{j=1}^{K}\mathrm{cov}({{\rm{V}}}_{j},{V}_{k})\end{array}$$

We then obtained the estimates for these parameters

9 $$\mathop{\eta }\limits^{\frown {}}=\frac{\sum _{k=1}^{K}\sum _{j=1}^{K}\mathrm{cov}({{\rm{V}}}_{k},{V}_{j})}{K}\,{\rm{and}}\,\hat{\nu }=\frac{2K}{\mathop{\eta }\limits^{\frown {}}}$$

We estimated the covariance cov(V*k*, *V*_*j*_) with a normal model^38^. In detail, let ${z}_{i}={{\rm{\Phi }}}^{-1}(1-{{\rm{p}}}_{i})$ be the transformed variable with standard normal distribution, where ${{\rm{\Phi }}}^{-1}(\cdot )$ is the inverse cumulative distribution function of the standard normal distribution. Then, the covariance to be estimated can be approximated by

10 $${\rm{Cov}}({{\rm{V}}}_{i},{V}_{j})\approx {{\rm{a}}}_{1}{\tilde{\rho }}_{ij}+{{\rm{a}}}_{2}{{\tilde{\rho }}_{ij}}^{2}+{{\rm{a}}}_{3}{{\tilde{\rho }}_{ij}}^{3}+{{\rm{a}}}_{4}{{\tilde{\rho }}_{ij}}^{4}$$

where ${a}_{1}=3.263119,\,{a}_{2}=0.709866,\,{a}_{3}=0.026589,\,{a}_{4}=-\,0.709866/{\rm{n}}$, *n* the sample size for obtaining *Z*_*i*_ and ${\hat{\rho }}_{ij}={\rm{Cor}}({Z}_{i},{Z}_{j})\,{\rm{and}}\,{\tilde{\rho }}_{ij}={\hat{\rho }}_{ij}(1+\frac{1-{{\hat{\rho }}_{ij}}^{2}}{2n-1})$. In the case of missing data, it is simple to ignore that data source and reduce the degree of freedom.

### Alternative p-values integration strategies

Besides Fisher’s method for *p*-values integration, we also selected three other methods for comparison. Given *K p*-values *p*_1_, …, *p*_*K*_ to be integrated.

#### minP

This method just takes the minimal *p*-values as integrated *p*-value with ${\tilde{p}}_{{\rm{minP}}}=\mathop{\min }\limits_{i=1,\mathrm{..}.,K}{p}_{i}$. It is easy to deal with missing data, as ignoring that data source.

#### Stouffer’s Z-score method

In this method, *p*-values are first transformed into z-scores with ${z}_{i}={{\rm{\Phi }}}^{-1}(1-{p}_{i})$ where ${\rm{\Phi }}(\cdot )$ is the standard normal cumulative distribution, then the overall meta-analysis statistic is calculated as $Z=\frac{{\sum }_{i=1}^{K}{z}_{i}}{\sqrt{K}}$, the integrated *p*-value is computed as ${\tilde{p}}_{{\rm{Stouffer}}}=1-{\rm{\Phi }}({\rm{Z}})$. Ignoring corresponding data source is enough to handle the missing data problem, as Fisher’s method does.

#### RRA

This method was proposed for aggregating ranking lists^35^, and it could be applied to *p*-values integration. Let ${p}_{(1)},\mathrm{..}.,{p}_{(K)}$ be a reordering of original *p*-values list, satisfying ${p}_{(1)}\le \mathrm{..}.\le {p}_{(K)}$, then the probability of m-th element of ordered *p*-values list generated from uniform distribution are more significant is

11 $${\beta }_{m}=\sum _{i=m}^{K}(\begin{array}{c}K\\ m\end{array}){p}_{(m)}^{i}{(1-{p}_{(m)})}^{K-m}$$

Then the integrated *p*-value is approximated as ${\tilde{p}}_{RRA}=K\mathop{\min }\limits_{i=1,\mathrm{.}.,K}{\beta }_{m}$, where *K* is used as Bonferroni correction. The same method is used to deal with missing data as above.

## Electronic supplementary material

Supplementary
