# Supplementary material for: Leveraging multiple genomic data to prioritize disease-causing indels from exome sequencing data
Source: Sci Rep. 2017 May 11;7:1804. doi: 10.1038/s41598-017-01834-w (PMC5431795; doi:10.1038/s41598-017-01834-w)
Supplement: Supplementary file 1 — Supplementary [file 41598_2017_1834_MOESM1_ESM.doc]

Supplementary materials for “**Leveraging multiple genomic data to prioritize disease-causing indels from exome sequencing data**”

### Mengmeng Wu^1,2^, Ting Chen ^1,2§^, Rui Jiang^1,3§^

^1^MOE Key Laboratory of Bioinformatics; Bioinformatics Division and Center for Synthetic & Systems Biology, TNLIST; Tsinghua University, Beijing 100084, China

^2^Department of Computer Science, Tsinghua University

^3^Department of Automation, Tsinghua University

^§^Corresponding author

Email addresses:

MMW: [wmm15@mails.tsinghua.edu.cn](mailto:wmm15@mails.tsinghua.edu.cn)

TC: [tingchen@tsinghua.edu.cn](mailto:tingchen@tsinghua.edu.cn)

RJ: [ruijiang@tsinghua.edu.cn](mailto:ruijiang@tsinghua.edu.cn)


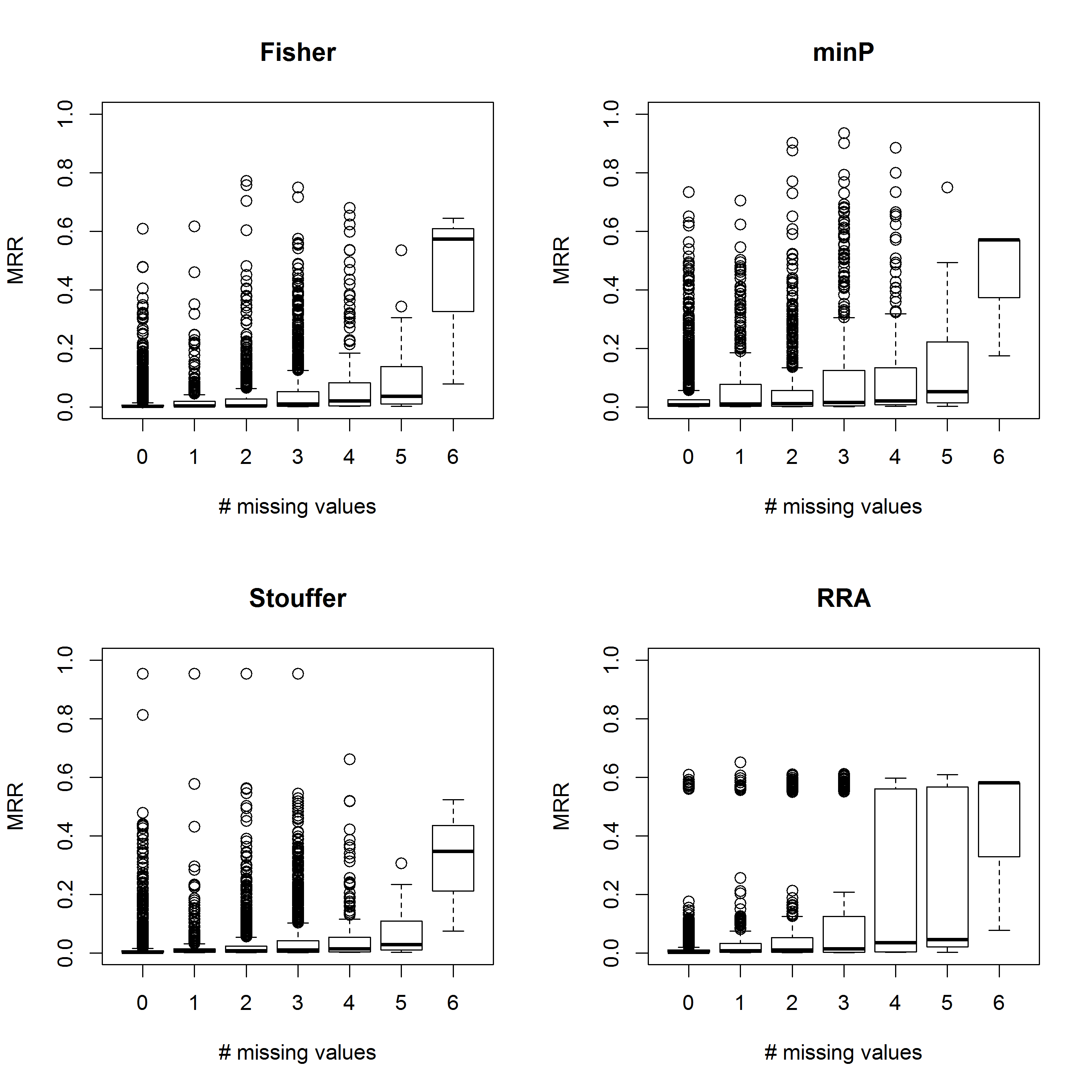


## Supplementary Figure 1. Robustness of different data fusion strategies in terms of missing data. The x axis denotes the number of missing data and the y axis denotes the rank ratios of indels with corresponding number of missing data.

##
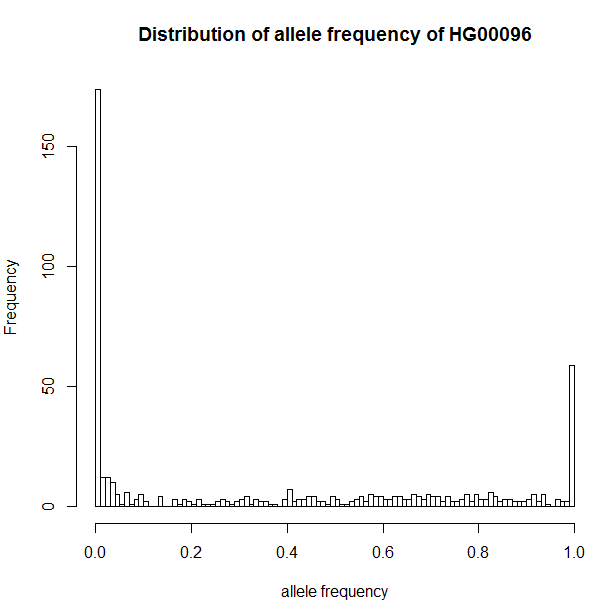


## Supplementary Figure 2. Allele frequency histogram of indels from HG00096 exome. The x axis denotes the allele frequency, which is defined as the proportion of KG individuals carrying the indel. The y axis denotes the number of indels with corresponding allele frequency.

##
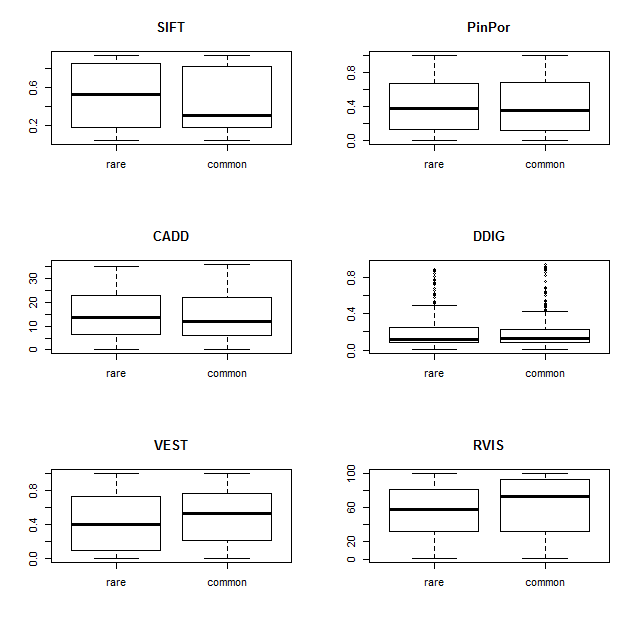


## Supplementary Figure 3. Functional prediction scores for common and rare indels of HG00096 exome. Each boxplot compares the distributions of different functional scores for common (allele frequency >=1%) and rare (allele frequency < 1%) of HG00096 individual exome.

## Supplementary Table 1. Summary statistics about data used in the prospective simulation study and corresponding performance. Abbreviations: ND (nonframeshift deletion); NI (nonframeshift insertion); FD (frameshift deletion); FI (frameshift insertion). First row denotes the number of indels for each indel subtype, and the second and third rows denote the MRRs and TOPs for each indel subtype.

|  | **ND** | **NI** | **FD** | **FI** |
| --- | --- | --- | --- | --- |
| **Number** | 59 | 17 | 501 | 237 |
| **MRR** | 0.9% | 1.4% | 2.1% | 4.2% |
| **TOP** | 55 | 16 | 477 | 218 |

## Supplementary Table 2. Comparison of different fusion strategies. Each entry denotes MRR or TOP of each alternative method. In summary, Fisher’s method and Stouffer’s method have the best performance, followed by minimal p-value method, and robust rank aggregation performs the worst. Abbreviations: ND (nonframeshift deletion); NI (nonframeshift insertion); FD (frameshift deletion); FI (frameshift insertion).

| Methods | MRR | | | | TOP | | | |
| --- | --- | --- | --- | --- | --- | --- | --- | --- |
|  | ND | NI | FD | FI | ND | NI | FD | FI |
| Fisher | 1.67% | 1.06% | 3.62% | 2.19% | 1,082 | 231 | 7,614 | 3,445 |
| minP | 3.89% | 3.11% | 7.43% | 5.98% | 1,014 | 209 | 7,367 | 3,216 |
| Stouffer | 2.21% | 1.40% | 3.56% | 2.82% | 1,122 | 240 | 8,108 | 3,592 |
| RRA | 5.40% | 3.09% | 8.80% | 5.83% | 1,071 | 233 | 7,484 | 3,390 |

## Supplementary Table 3. Performance on 1000 Genomes Project individual exome data. The first column records the individual ID and corresponding population, and the following columns record the number of different indels, the MRR and the TOP for different indels. Abbreviations: GBR, British in England and Scotland; FIN, Finnish in Finland; CHS, Southern Han Chinese, China; PUR, Puerto Rican in Puerto Rico; IBS, Iberian populations in Spain; ESN, Esan in Nigeria; CEU, Utah residents with Northern and Western European ancestry; CHB, Han Chinese in Beijing, China; LWK, Luhya in Webuye, Kenya; YRI, Yoruba in Ibadan, Nigeria; ND (nonframeshift deletion); NI (nonframeshift insertion); FD (frameshift deletion); FI (frameshift insertion).

| Individual (population) | Number | | | | MRR | | | | TOP | | | |
| --- | --- | --- | --- | --- | --- | --- | --- | --- | --- | --- | --- | --- |
|  | ND | NI | FD | FI | ND | NI | FD | IFI | ND | NI | FD | FI |
| HG00096 (GBR) | 115 | 79 | 211 | 117 | 3.07% | 2.28% | 5.31% | 3.80% | 479 | 111 | 2,992 | 1,343 |
| HG00171 (FIN) | 58 | 59 | 118 | 95 | 2.92% | 2.24% | 5.23% | 3.58% | 512 | 116 | 3,243 | 1,437 |
| HG00403 (CHS) | 60 | 53 | 115 | 92 | 2.62% | 1.88% | 4.85% | 3.29% | 513 | 116 | 3,243 | 1,442 |
| HG00551 (PUR) | 64 | 56 | 103 | 92 | 2.53% | 1.81% | 4.67% | 3.17% | 515 | 117 | 3,273 | 1,459 |
| HG01500 (IBS) | 63 | 62 | 117 | 106 | 2.77% | 2.07% | 4.91% | 3.40% | 512 | 116 | 3,246 | 1,439 |
| HG02922 (ESN) | 74 | 64 | 133 | 93 | 2.55% | 1.78% | 4.65% | 3.15% | 508 | 116 | 3,232 | 1,436 |
| NA06984 (CEU) | 50 | 56 | 107 | 100 | 2.88% | 2.22% | 5.02% | 3.52% | 514 | 116 | 3,260 | 1,452 |
| NA18525 (CHB) | 60 | 51 | 120 | 80 | 2.49% | 1.83% | 4.77% | 3.18% | 515 | 117 | 3,274 | 1,461 |
| NA19017 (LWK) | 77 | 62 | 126 | 102 | 2.46% | 1.76% | 4.49% | 3.03% | 511 | 116 | 3,237 | 1,440 |
| NA19092 (YRI) | 80 | 57 | 134 | 107 | 2.78% | 2.09% | 4.87% | 3.38% | 506 | 116 | 3,204 | 1,429 |
| Average | 70 | 60 | 128 | 98 | 2.71% | 1.99% | 4.88% | 3.35% | 509 | 116 | 3,220 | 1,434 |
